# Urinary Biomarkers α-GST and π-GST for Evaluation and Monitoring in Living and Deceased Donor Kidney Grafts

**DOI:** 10.3390/jcm8111899

**Published:** 2019-11-07

**Authors:** Shadi Katou, Brigitta Globke, M. Haluk Morgul, Thomas Vogel, Benjamin Struecker, Natalie Maureen Otto, Anja Reutzel-Selke, Marion Marksteiner, Jens G. Brockmann, Andreas Pascher, Volker Schmitz

**Affiliations:** 1 Department of General, Visceral and Transplantation Surgery, Universitätsklinikum Münster, 48189 Münter, Germany; haluk.morguel@ukmuenster.de (M.H.M.); thomas.vogel@ukmuenster.de (T.V.); benjamin.struecker@ukmuenster.de (B.S.); jens.brockmann@ukmuenster.de (J.G.B.); andreas.pascher@ukmuesnter.de (A.P.); schmitz.volker@icloud.com (V.S.); 2 Charité – Universitätsmedizin Berlin, corporate member of Freie Universität Berlin, Humboldt-Universität zu Berlin, and Berlin Institute of Health, Department of Surgery CCM|CVK, 10117 Berlin, Germany; brigitta.globke@charite.de (B.G.); anja.selke@charite.de (A.R.-S.); marion.marksteiner@charite.de (M.M.); 3 Department of Nephrology and Internal Intensive Care Medicine, Charité Universitätsmedizin Berlin, Campus Virchow Klinikum, 10117 Berlin, Germany; natalie.otto@charite.de

**Keywords:** kidney transplantation, urinary biomarkers, α-GST, π-GST, acute rejection, delayed graft function, nephrotoxicity

## Abstract

The aim of this study was to analyze the value of urine α- and π-GST in monitoring and predicting kidney graft function following transplantation. In addition, urine samples from corresponding organ donors was analyzed and compared with graft function after organ donation from brain-dead and living donors. Urine samples from brain-dead (*n* = 30) and living related (*n* = 50) donors and their corresponding recipients were analyzed before and after kidney transplantation. Urine α- and π-GST values were measured. Kidney recipients were grouped into patients with acute graft rejection (AGR), calcineurin inhibitor toxicity (CNI), and delayed graft function (DGF), and compared to those with unimpaired graft function. Urinary π-GST revealed significant differences in deceased kidney donor recipients with episodes of AGR or DGF at day one after transplantation (*p* = 0.0023 and *p* = 0.036, respectively). High π-GST values at postoperative day 1 (cutoff: >21.4 ng/mg urine creatinine (uCrea) or >18.3 ng/mg uCrea for AGR or DGF, respectively) distinguished between rejection and no rejection (sensitivity, 100%; specificity, 66.6%) as well as between DGF and normal-functioning grafts (sensitivity, 100%; specificity, 62.6%). In living donor recipients, urine levels of α- and π-GST were about 10 times lower than in deceased donor recipients. In deceased donors with impaired graft function in corresponding recipients, urinary α- and π-GST were elevated. α-GST values >33.97 ng/mg uCrea were indicative of AGR with a sensitivity and specificity of 77.7% and 100%, respectively. In deceased donor kidney transplantation, evaluation of urinary α- and π-GST seems to predict different events that deteriorate graft function. To elucidate the potential advantages of such biomarkers, further analysis is warranted.

## 1. Introduction

Kidney transplantation is by far the best therapeutic option for patients with end-stage renal disease (ESRD). After transplantation, the main challenges, besides surgical complications, are acute graft rejection, delayed graft function, and adverse effects of immunosuppressants [1]. Acute graft rejection still occurs in up to 25% of recipients and is a significant prognostic factor for long-term graft survival [2]. The improvements of immunosuppressive drugs have turned transplantation into a safe and widely predictable therapy; however, many of the agents used today still contribute to graft failure due to their nephrotoxic potential [3]. Delayed graft function, defined by the need for dialysis within the first week after transplantation and mainly caused by acute tubular necrosis, is mostly due to long ischemia times, advanced donor age, and comorbidities [4,5]. Recognizing the cause of graft dysfunction may be challenging, yet immediate diagnosis and therapy are essential for optimal graft survival. 

The signs of graft dysfunction are decreased diuresis and impaired creatinine blood levels. Monitoring immunosuppressive drugs and their toxicity through serum levels is of limited value, since the difference between therapeutic and toxic levels is not fixed [6]. In order to define the pathomechanism of graft dysfunction, a graft biopsy is required in most cases. However, this is an invasive procedure and risks associated complications endangering the transplanted kidney [7].

α- and π-GST, which are specifically present in the kidney tubules, are two isotypes of the glutathione-S-transferases. Beyond their biochemical differences, they are also located in different parts of the tubule system [8]. α-GST is found in cells of the proximal tubules, which are predominantly affected by ischemia time and nephrotoxic substances. π-GST is located in distal tubules, which are damaged during acute graft rejection [9]. Their release into the urine as a result of cell damage gives an accurate prediction of the impaired part of the tubules system and therefore the underlying cause of graft dysfunction [10,11]. Analyses of α- and π-GST have been reported to be promising for discriminating between the different causes of graft dysfunction [11,12,13].

The aim of our study was to determine the value of measuring α- and π-GST concentrations in urine as biomarkers for monitoring graft function and predicting postoperative events in the first week after transplantation in living and deceased donor kidney transplantation.

## 2. Experimental Section

### 2.1. Samples and Data Collection

This study was approved by the Ethics Committee of Berlin’s Charité University Hospital (EA2/137/10). We prospectively analyzed blood and urine samples as well as demographic data from 160 patients: 30 brain-dead donors and their 30 corresponding recipients; and 50 living kidney donors as well as their 50 corresponding living donor kidney recipients. All surgeries were carried out at the Department of General, Visceral and Transplantation surgery of Charité University Hospital, Berlin. Machine perfusion was not performed for any kidney allograft in this study. Except for brain-dead donors, all patients were followed during the first week after surgery. Blood and urine samples were collected at the following time points: day 0, day 1, day 3, day 5, and day 7. Samples from brain-dead organ donors were obtained on day 0, the day of the organ donation surgery. Urine samples were collected from recipients after transplantation through an externalized uretero-vesico-cutaneous stent and therefore exclusively reflected the α- and π-GST content of the transplanted grafts.

### 2.2. Immunosuppression Events and Subgroups

**Figure 1:**
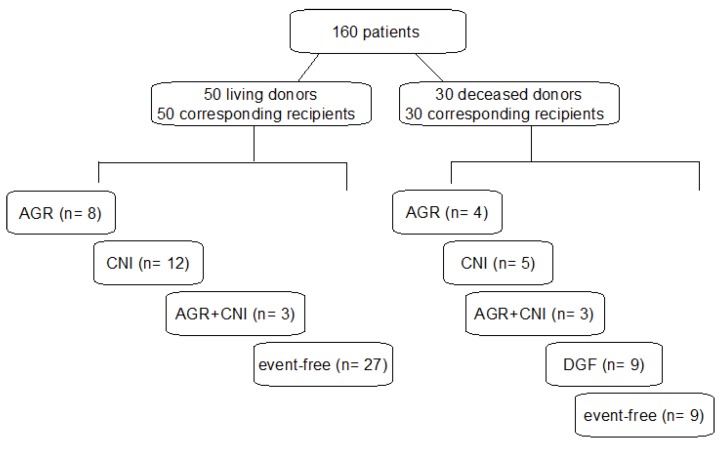
Patients, groups, and subgroups.

All recipients received a triple immunosuppressant consisting of prednisolone, mycophenolate mofetil (MMF), and a calcineurin inhibitor. All deceased donor kidney recipients received tacrolimus, whereas living donor kidney recipients were treated with either tacrolimus or cyclosporine. Recipients were divided into subgroups according to the events in the first postoperative week: acute graft rejection (AGR, G1), calcineurin-induced nephrotoxicity (CNI, G2), both acute kidney rejection and calcineurin-induced nephrotoxicity (AGR + CNI, G3), delayed graft function (DGF, G4), and event-free (healthy, G5) subgroup (Figure 1). An acute graft rejection was confirmed by graft biopsy and classified according to the BANFF criteria. Calcineurin-induced nephrotoxicity was defined by serum levels of agents (tacrolimus >15 ng/mL, cyclosporin >250 ng/mL). It is worth noting that delayed graft function, characterized by the need for dialysis in the first week after transplantation, was not recorded in any of the living donor recipients. In case dialysis was required due to a known graft-damaging event such as AGR or CNI, those patients were enrolled in subgroups of primary cause and not defined as DGF. Recipients with no signs for any of the above events were considered healthy and were used as our control group.

### 2.3. GST Analysis and Statistics 

Urinary α- and π-GST values were measured using a commercially available ELISA test kit provided by Argutus Medical Ltd. (Dublin, Ireland). To consider the physiological differences in urine concentrations, α- and π-GST values were standardized to urine creatinine. The resulting unit for GST was ng/mg uCrea. Reference ranges of urinary α- and π-GST were determined as recommended from the measured GST values in the healthy population of this study group. For this purpose, the α- and π-GST values in the urine of healthy living donors were considered before donor nephrectomy. The reference interval for α-GST is 2.7–7.6 ng/mg uCrea and for π-GST 4.1–13 ng/mg uCrea in this study.

For statistical analysis, GraphPad Prism 6 (GraphPad Software, San Diego, CA, USA) was used. Quantitative data are given as the mean and standard deviation. To compare normally distributed variables, *t*-tests such as Mann‒Whitney and Wilcoxon were performed. For the comparison of multiple variables, we used two-way ANOVA; here we applied Tukey and Holm‒Sidak tests for post hoc analysis of the subgroups. The area under the curve was calculated in ROC analysis and a log-rank (Mantel‒Cox) test was performed in survival analysis. A *p*-value less than 0.05 was defined as significant.

## 3. Results

### 3.1. Demographic Data

**Figure 2:**
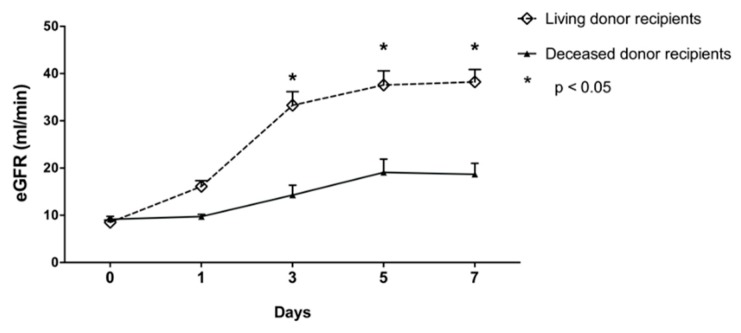
eGFR comparison between deceased and living donor recipients before and during the first week after transplantation.

There were no differences regarding gender and BMI between the patient groups. The mean eGFR before graft recovery or transplantation (d0) did not differ between the groups. Recipients of deceased donor kidneys were significantly older than those of living donors (58 ± 13 and 48 ± 15 respectively, *p* = 0.006), whereas the donor’s age was not different. Cold as well as warm ischemic time were significantly higher in the deceased donor group (both *p* < 0.0001). The demographic data of donors and recipients are given in Table 1. The increase in the eGFR of recipients after transplantation was, as expected, more noticeable in living donation scenarios (Figure 2).

### 3.2. α- and π-GST in Recipients

**Figure 3:**
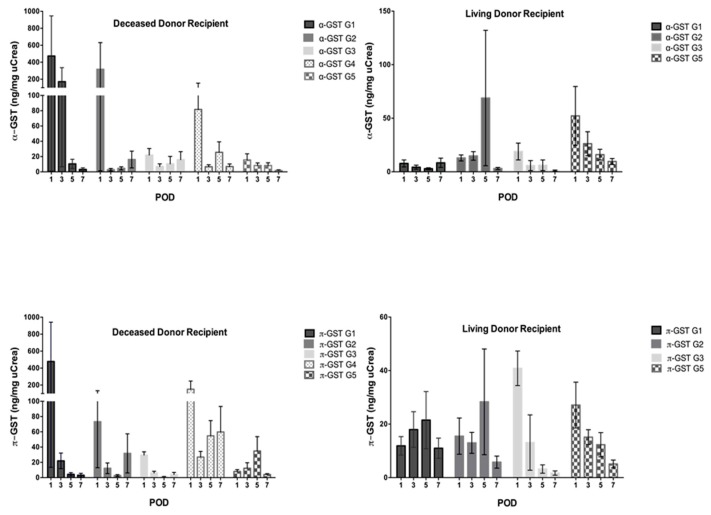
Urinary α- and π-GST levels in subgroups of deceased and living donor recipients during the first week after transplantation; G1: Acute graft rejection (AGR), G2: Calcineurin-induced nephrotoxicity (CNI), G3: Simultaneous acute graft rejection and calcineurin-induced nephrotoxicity (AGR + CNI), G4: Delayed graft function (DGF), G5: Event-free (control).

Neither α- nor π-GST correlated with age, BMI, and cold or warm ischemic time in any group. However, both GST isoenzymes correlated with renal function in living donors and in the healthy recipients subgroup. In deceased donor recipients and living donor recipients we observed acute rejection in four (13.4%) and eight patients (16%), calcineurin-induced nephrotoxicity in five (16.7%) and 12 (24%) patients, and both simultaneously in three (10%) and three (6%) patients, respectively. Delayed graft function occurred only with deceased donors (nine patients, 30%). Patients with an uneventful postoperative course in the deceased donor recipient group numbered nine (30%) and, in the living donor recipient group, 27 (54%). Both α- and π-GST were significantly elevated at 1st postoperative day (POD) in deceased donor recipients, with acute rejection when compared with the corresponding healthy subgroup (α-GST: Mean 473.5 ± 818 vs. 15.6 ± 21.2 ng/mg uCrea, *p* = 0.0094; π-GST: mean 477.8 ± 804 vs. 8 ± 6.4 ng/mg uCrea, *p* = 0.0023). In living donor recipients, only π-GST showed an increase, reaching a peak at day 5 (mean 21.5 ± 28.2 ng/mg uCrea); however, there was no significant difference between this group and the healthy subgroup. In patients with CNI toxicity, α-GST performed better in both recipient groups; in deceased donor recipients, the mean was 316 ± 704.5 ng/mg uCrea at 1 POD and was easily discriminated from the uneventful subgroup (*p* = 0.06). Also, in the living donor group, a rise of α-GST to 68.9 ± 219 ng/mg uCrea was noted at day 5, when CNI toxicity occurred. When both acute rejection and CNI toxicity were recorded, neither α- nor π-GST was able to distinguish those patients; however, it is worth mentioning that the number of subjects in this subgroup was very low in our study. In the case of delayed graft function, present only in deceased donor recipients in our study, α- as well as π-GST were elevated in the urine, with means at 1st POD of 81.5 ± 201.3 and 151.6 ± 270.6 ng/mg uCrea, respectively. Only π-GST levels proved significant when compared to the control subgroup (*p* = 0.036) (Figure 3).

**Figure 4:**
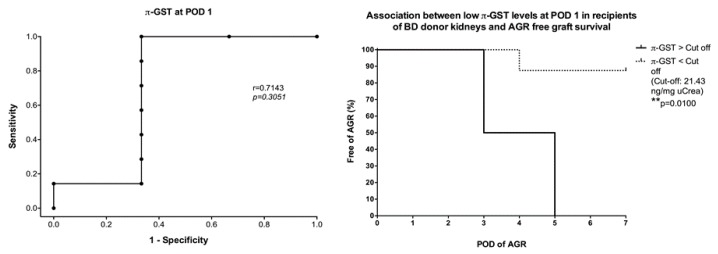
Correlation between urinary π-GST levels in recipients of brain-dead donors’ grafts on POD 1, and probability of graft survival without acute graft rejection (AGR) during the first week after transplantation.

**Figure 5:**
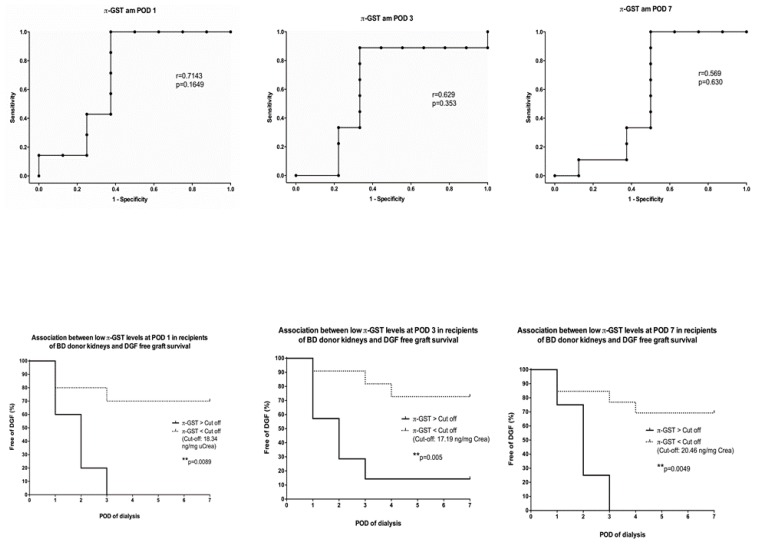
Correlation between urinary π-GST levels in recipients of brain-dead donors’ grafts on POD 1, 3, and 7; and probability of graft survival without delayed graft function (DGF) during the first week after transplantation.

Furthermore, we performed ROC curve and survival analysis on the most outstanding biomarkers at a specific point in the study. π-GST showed the most promising results in deceased donor recipients with acute graft rejection and delayed graft function at day 1 after transplantation. Patients who developed a biopsy confirmed acute graft rejection within the first week after transplantation had significantly higher levels of urinary π-GST at POD 1. With an estimated cutoff of 21.4 ng/mg uCrea, π-GST was able to distinguish the occurrence of AGR from a rejection-free course with 100% and 66.6% sensitivity and specificity, respectively (Figure 4). Similar reliability for π-GST was observed in the DGF subgroup; however, the estimated cutoff at POD 1 was slightly lower at 18.3 ng/mg uCrea (sensitivity, 100%; specificity, 62.6%). Higher urinary π-GST levels could be seen in recipients with delayed graft function; this was observed at POD1, POD 3 as well as POD 7. π-GST was not able to differentiate between the causes of graft dysfunction in the early postoperative period (Figure 5). 

### 3.3. α- and π-GST in Donors

**Figure 6:**
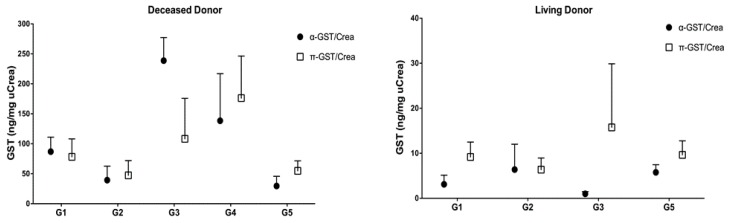
Urinary α- and π-GST levels in deceased donors before transplantation; G1: Acute graft rejection (AGR), G2: Calcineurin-induced nephrotoxicity (CNI), G3: Simultaneous acute graft rejection and calcineurin-induced nephrotoxicity (AGR+CNI), G4: Delayed graft function (DGF), G5: Event-free (control).

**Figure 7:**
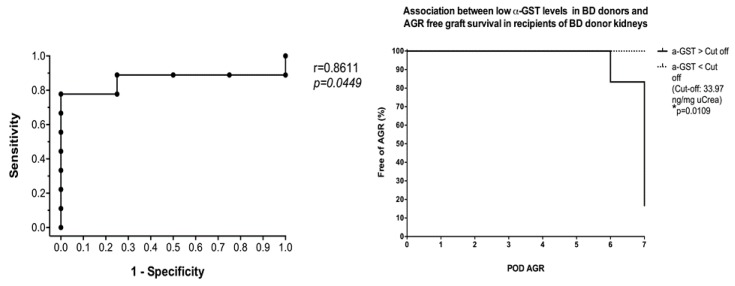
Correlation between urinary α-GST levels in brain-dead donors before transplantation and probability of graft survival without acute graft rejection in corresponding recipients during the first week after transplantation.

α- and π-GST showed interesting results when measured in deceased donor urine before organ harvesting as they were elevated in those with poorer graft function after transplantation, and seemed to predict a foreseeable event such as acute rejection or delayed graft function, which was 3- to 8-fold more likely to occur than in those recipients with an uneventful course of treatment. α-GST stood out in the subgroup with both acute rejection and CNI toxicity (*p* = 0.02), while α- and π-GST were remarkably higher (but not significantly so) in the DGF subgroup compared to the control group (Figure 6). As for the predictive value of GST when measured in donor urine, α-GST stood out with significant results in the AGR subgroup when ROC curve analysis was performed, and a cutoff value of >33.97 ng/mg uCrea was calculated (AUC, 0.86; sensitivity, 77.7%; specificity, 100%). Based on the donor’s urinary α-GST alone, all four renal grafts from deceased donors that showed acute rejection in recipients were distinguished from those who had an AGR-free course in the survival curve analysis (*p* = 0.0109) (Figure 7).

On the other hand, urinary GST in living donors showed no differences between subgroups and the corresponding control group, and therefore failed to predict future events in recipients.

### 3.4. Six- and 12-Months Graft Survival 

We followed up recipients of the subgroups G1 (AGR) and G4 (DGF) at six and 12 months after transplantation, as correlations of α- and π-GST in those cohorts showed the most promising results. However, in subgroup G1 only one patient out of four lost the graft due to recruiting nephritis; in subgroup G4 all three grafts were lost due to death not associated with graft function (cancer or cardiac arrest).

## 4. Discussion

The results of our prospective study, evaluating urinary α- and π-GST in deceased as well as living kidney donors and their corresponding recipients as biomarkers for graft quality and function, suggest the potential value of these enzymes. Previous studies showed the ability of urinary α- and π-GST to predict acute renal damage in kidney graft recipients and demonstrated a release of these enzymes in malfunctioning grafts [10,11,12,13,14,15]. However, none of these groups has compared the course of α- and π-GST in donors as well as in corresponding recipients. In addition, we investigated the differences in the markers in two settings, brain-dead/deceased and living organ donation. Our analyses reveal that the determination of urinary π-GST concentration in deceased donor recipients, especially on the first day after graft transplantation, could be valuable and indicative of kidney allograft function and survival without AGR or DGF. Secondly, we found higher concentrations of α- and π-GST in the urine of deceased kidney donors, whose grafts performed poorly in the corresponding recipients; α-GST was able to predict AGR before transplantation. A determination between the causes of impaired allograft function could not be reached by assessing urinary α- and π-GST alone, though it is unlikely that in the complex setting of transplantation a single biomarker will reliably distinguish between the pathogenesis of multifactorial elements; therefore, the proposed markers should be seen as an useful tool in addition to established methods.

Research studies investigating α- and π-GST in living donation are extremely limited: in our review of the literature we only found one publication on this issue, and this concerned liver rather than kidney transplantation [16]. Our findings showed lower concentrations of α- and π-GST in the urine of living donor kidney recipients than in that of deceased donor kidney recipients. This was observed in almost all subgroups, and especially on POD 1. Except for a notable, yet insignificant, rise of π-GST in living donor recipients with AGR when compared to the control group, our results find no further significances of urinary α- and π-GST in living donor recipients when harmful events occurred. Considering the superior organ quality and logistics in living donation transplantation, as demonstrated by over half of living donor grafts surviving the first week after transplantation event-free compared to 30% of deceased donor grafts, lower urinary concentrations of α- nor π-GST in living donor transplantation are to be expected. Daemen et al. found a correlation between α-GST and warm ischemic time in grafts from donation after cardiac death [17]. In our study neither α- nor π-GST had a proven correlation with ischemic time, although it should be noted that a different type of graft was investigated in the work mentioned. 

The toxic effect of CNI agents on renal grafts and its association with the excretion of α-GST has been described in the past [10,18]. Our results failed to indicate such a correlation in deceased or living donor recipients. This might be due to our definition of the toxic range of CNI serum levels. Therapeutic and toxic serum levels of several drugs and especially immunosuppressants have been known to be inconsistent and even overlapping [6,19]. In the 1990s, serum levels of tacrolimus in the early period after transplantation were suggested to be below 20 ng/mL in order to avoid side effects, whereas later on levels above 15 ng/mg were proven to be associated with a higher risk of toxicity [20,21]. On the other hand, the risk of acute rejection is significantly higher when there are low concentrations of the agent [22]. A helpful step would be to find the toxic serum level of immunosuppressants that is agreed upon by transplant communities; currently, despite all efforts, this varies significantly between transplant centers. Another concerning factor is the design of the study, which did not include daily and therefore more precise surveillance on that matter. In order to investigate the correlation between toxic exposure to immunosuppressants and the excretion of GST into the urine, a closer observation with more frequent sample collection is required.

DGF was observed only in deceased donor recipients in our study. It occurred with an incidence of 30%, which is similar to the findings of a recent work by Willicombe et al. [23]. Risk factors and characteristics of donors and recipients associated with DGF such as cold ischemic time and donor age have been established in previous publications [4,5,24]. However, taking these factors into consideration, it is to be expected that DGF is less common in living donation. It has been demonstrated that α-GST excretion would be increased in the case of DGF due to its location in the renal tubular system and the association between DGF and proximal tubular necrosis [8,25]. On the other hand, π-GST has been shown to be of predictive value in terms of the need for dialysis in a publication by Seabra et al. including 245 patients with acute kidney injury [26]. Hall et al. investigated α- and π-GST in a perfusate solution during machine perfusion of kidney allografts from deceased donors, and suggested an independent association between π-GST and DGF [27]. Our findings demonstrated the consistent significance of urinary π-GST in differentiating between DGF and normally functioning grafts when measured in deceased donor recipients. This was observed at several time points of the study and had a strong power of sensitivity and specificity. Little is known about the behavior of the proteins under dialysis, so it is unclear whether α-GST is more dialyzable than π-GST or the other way round. The sample collection from patients undergoing dialysis in our study did not occur with respect to dialysis time, which should be seen as another possible disturbance factor. 

Further limitations of this study are the small number of patients in certain subgroups and the overall high standard deviations. We distinguished well between the causes of impaired graft function and took into consideration simultaneous events. The time frame of our observation was limited in that it focused only on the first week after transplantation. We believe that multiple serial samples and an extended study design would be beneficial in future projects.

## 5. Conclusions

In summary, the elevation of urinary π-GST in deceased donor kidney recipients at day 1 after kidney transplantation could be a helpful monitoring parameter, in addition to urinary output and serum creatinine, to determine graft function in recipients. It might be indicative of acute rejection or a need for dialysis. The measuring of urinary α- and π-GST should also be considered in deceased donors as this seems to be of predictive value in terms of graft outcome and might help with assessing allograft quality. Our findings reveal an association between urinary α-GST in deceased donors and AGR in corresponding recipients. Thus, further investigation of α- and π-GST in a larger population and daily sample collection should be considered. Although urinary α- and π-GST in living kidney donation showed no relevant correlation with harmful events in our analyses, this is, to the best of our knowledge, the first study demonstrating differences in biomarkers between deceased and living kidney donation, so subsequent investigations will be needed in order to confirm or contradict our findings.

## Figures and Tables

**Table 1:** Demographic data of donors and recipients.

	Deceased Donor Grafts	Living Donor Grafts	*p* Value
Donor	30	50	
Age (years)	58 ± 15	53 ± 10	n.s.
Sex (male/female)	11/19	22/28	n.s.
BMI	27.3 ± 6.5	25.3 ± 3.2	n.s.
eGFR, d0 (mL/min)	96 ± 39	97 ± 18	n.s.
Diuresis in last hour (mL)	152 ± 81	-	
Recipient	30	50	
Age (years)	58 ± 12	47 ± 15	<0.05
Sex (male/female)	22/8	35/15	n.s.
BMI	26.4 ± 4	25.8 ± 4.9	n.s.
eGFR, d0 (mL/min)	9 ± 3	8 ± 4	n.s.
Primary disease			
Glomerulonephritis	9	24	n.s.
Hypertensive nephrosclerosis	7	9	n.s.
Polycystic kidney disease	6	2	n.s.
Autoimmune disease	2	5	n.s.
Diabetes	2	3	n.s.
Urologic disease	2	2	n.s.
Calcineurin-induced nephrotoxicity	0	2	n.s.
Others	2	3	n.s.
Dialysis			
Hemodialysis	29	28	n.s.
Peritoneal dialysis	1	3	n.s.
No dialysis	0	19	<0.05
Cold ischemia time (minutes)	613 ± 269	193 ± 62	<0.05
Warm ischemia time (minutes)	34 ± 11	23 ± 7	<0.05

BMI, body mass index; eGFR, estimated glomerular filtration rate; n.s., not significant.
